# Effects of acute critical illnesses on the performance of interferon-gamma release assay

**DOI:** 10.1038/srep19972

**Published:** 2016-01-25

**Authors:** Chun-Ta Huang, Sheng-Yuan Ruan, Yi-Ju Tsai, Ping-Hung Kuo, Shih-Chi Ku, Pei-Lin Lee, Lu-Cheng Kuo, Chia-Lin Hsu, Chun-Kai Huang, Ching-Yao Yang, Ying-Chun Chien, Jann-Yuan Wang, Chong-Jen Yu

**Affiliations:** 1 Department of Internal Medicine, National Taiwan University Hospital, Taipei 100, Taiwan; 2 Department of Traumatology, National Taiwan University Hospital, Taipei 100, Taiwan; 3 Graduate Institute of Clinical Medicine, National Taiwan University, Taipei 100, Taiwan; 4 School of Medicine, College of Medicine, Fu-Jen Catholic University, New Taipei 242, Taiwan; 5 Center of Sleep Disorder, National Taiwan University Hospital, Taipei 100, Taiwan

## Abstract

Performance of interferon-gamma release assays (IGRAs) is influenced by preanalytical, laboratory and host factors. The data regarding how critical illnesses influence IGRA results are limited. This study aimed to investigate IGRA performance among critically ill patients. Patients admitted to intensive care unit (ICU) were prospectively enrolled, and underwent QuantiFERON-TB Gold In-Tube testing on admission and discharge. The associations between patient factors and IGRA results were explored. In total, 118 patients were included. IGRA results on admission were positive, negative and indeterminate for 10(9%), 36(31%) and 72(61%) patients. All indeterminate results were due to a low mitogen response. Indeterminate results were associated with higher disease severity and lower serum albumin levels. Ninety(76%) patients survived to ICU discharge and had repeat IGRA testing 13.3 ± 10.1 days after first ones. Of those, 43(48%) had indeterminate results, and no IGRA conversion or reversion was observed. The majority (35/51, 69%) of ICU survivors with initial indeterminate results still had indeterminates on follow-up testing. Acute critical illnesses exert a significant impact on IGRA performance and a high proportion of indeterminate results was seen in ICU patients. This study highlights limitation of IGRAs in the critically ill and judicious selection of patients to be tested should be considered.

One-third of the individuals worldwide are infected with *Mycobacterium tuberculosis* (MTB)^1^. Among them, immunocompetent subjects have a 5–10% lifetime risk of progressing to active tuberculosis (TB). Thus, latent TB infection (LTBI) serves as a significant reservoir of future epidemics. Identification and treatment of LTBI is a key element in post-2015 strategy for global TB control^2^. The tuberculin skin test (TST) and interferon-gamma (IFN-γ) release assays (IGRAs) are currently available methods for diagnosis of LTBI. The TST has been the most widespread used test for detecting LTBI since a century ago^3^. Until recently, IGRAs arise as a promising alternative to the TST because of their equivalent sensitivity and improved specificity^4^,^5^. IGRAs are functional assays measuring T cell response to MTB-specific antigens in either whole blood or peripheral blood-derived mononuclear cells^5^. IGRAs also have advantages of an objective readout, no need for revisit and no boost effect compared to the TST^4^. In light of this, IGRAs have been incorporated into international guidelines for LTBI screening and diagnosis in several countries, either as a confirmatory test for a positive TST or as a substitute for the TST^6^.

Despite these logistical advantages, many issues regarding IGRAs, such as suboptimal reproducibility, unknown prognostic value and limited interpretive criteria, need to be settled^7^. The causes of considerable IGRA variability are far from fully understood. It has been shown that assay, preanalytical and analytical factors all have an impact on reproducibility of IGRA results^8^,^9^,^10^. Even a small change in blood volume or the extent of tube shaking may significantly influence IGRA performance^11^. These emphasize the importance of assay standardization and appropriate quality control. Additionally, IGRA performance may be altered because of host biological and immunological variations; certain chronic illnesses or conditions like age, malnutrition, lymphocytopaenia, human immunodeficiency virus (HIV) infection, malignancy and renal dysfunction are recognized ones^12^,^13^,^14^. Acute febrile illnesses or dysfunction of organs are associated with altered immune reactions^15^,^16^,^17^, and they may theoretically have effects on the performance and reproducibility of IGRAs. However, little is known about how acute insults affect IGRA results. Clinically, such data are essential because diagnosis and treatment decisions could be impacted by testing results. In this way, physicians would better realize the limitations in applying IGRAs.

In the present study, we aimed to investigate IGRA performance among patients suffering from acute critical illnesses. In addition, we explored patient factors, which may influence the IGRA results.

## Methods

### Study population

This prospective observational study was conducted in the National Taiwan University Hospital (NTUH). The study received approval from the Research Ethics Committee of the NTUH (201308080RIND) and written informed consent has been obtained from all patients before the study. In addition, the study was conducted in accordance with the amended Declaration of Helsinki. From May 2014 to December 2014, all critically ill patients were eligible for this study if they were aged 20 years or more and provided written informed consent. To study the effects of acute critical illnesses on the performance and reproducibility of IGRAs, patients were excluded if they were admitted from the ward or referred from another hospital, had hospitalizations in past 3 months, were not expected to survive or stay in the intensive care unit (ICU) longer than 24 hours, or had HIV infection.

### Data collection

Variables retrieved included demographics, body mass index, comorbidities, history of prior TB, Bacillus Calmette-Guerin (BCG) vaccination status, diagnosis of acute illnesses, blood testing results, events during the ICU stay and patient outcomes. BCG vaccination status was assessed by examination for the presence of BCG scar and information from the patients. Acute physiology and chronic health evaluation (APACHE) II scores were calculated^18^.

### Blood sampling and assays

Venous blood samples were obtained on ICU admission and again before discharge. To minimize preanalytical variability, blood sampling was done by a single technician, and samples were transported within 1 hour to the laboratory and immediately processed. The QuantiFERON-TB Gold In-Tube (QFT-GIT) test (Qiagen, Carnegie, Australia) was applied in this study. The assays were performed strictly according to the manufacturer’s instructions^19^. To avoid laboratory variability, the QFT-GIT assay was done by the same experienced technician who was blinded to the clinical information. Positive, negative and indeterminate results were defined per manufacturer^20^. ^20^IGRA conversion was defined as a negative result at baseline and a positive result on subsequent testing; IGRA reversion was defined as a change from a positive QFT-GIT result to a negative QFT-GIT result according to CDC guidelines^21^. Because the upper limit of accuracy in QFT-GIT assays was an IFN-γ level of 10 international units (IU)/ml, all values greater than 10 IU/ml were truncated at 10 IU/ml for the analysis.

### Statistical analysis

Analyses were conducted employing statistical software SPSS (version 15.0, SPSS Inc., Chicago, IL). Data were presented as mean ± standard deviation, number or number (%). Inter-group comparisons were analysed using the Student t test or one-way analysis of variance for continuous variables, and χ^2^ or Fisher’s exact test for categorical variables. The multivariate logistic regression analysis was performed to identify independent factors to affect the QFT-GIT results. All P values were two-tailed and a P value of <0.05 denoted statistical significance.

## Results

### Patients

**Figure 1:**
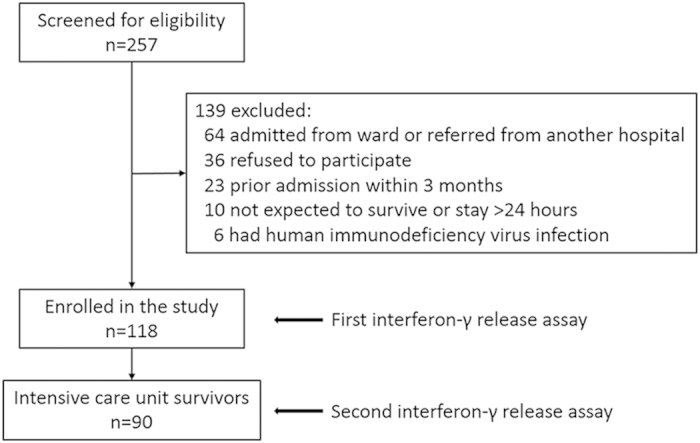
Study flow diagram.

Between May and December 2014, 257 ICU patients were screened for eligibility and finally a total of 118 patients participated in the study (Fig. 1). Characteristics of the study subjects are listed in Table 1. The mean age was 62 years and 69 (59%) were men. The mean APACHE II score on ICU admission was 21. Previous BCG vaccination was confirmed in 105 (89%) patients and 10 (8%) study subjects had active or prior history of TB. The ICU and hospital mortality rates were 24% and 36%, respectively.

### QFT-GIT results

Overall, 10 (9%) of the patients had a positive QFT-GIT result and 36 (31%) of the study subjects had a negative QFT-GIT result on ICU admission. All indeterminate results (72/118, 61%) were due to a low mitogen response. Patients with indeterminate QFT-GIT results tended to have higher ICU mortality than those with interpretable ones (20/72, 28% vs. 8/46, 17%; p = 0.196).

The comparison of the proportion of positive, negative and indeterminate QFT-GIT results between patient groups with different disease severity is shown in Table 2. Patients with a higher severity score had worse ICU and hospital outcomes. Indeterminate QFT-GIT findings occurred more often among patients with higher APACHE II scores compared with those with lower scores. Moreover, the mitogen response diminished as patients’ disease severity increased.

### Variability of QFT-GIT results

Ninety (76%) patients survived to ICU discharge and had paired QFT-GIT results (Table 3). The interval between first and second IGRA testing was 13.3 ± 10.1 days. On ICU discharge, 43 (48%) ICU survivors had an indeterminate QFT-GIT result. Of note, 51 patients with an initial indeterminate result survived to be discharged from the ICU and 35 (69%) of them still had an indeterminate result. No IGRA conversion or reversion was found in this study. There was no difference in the time interval between paired QFT-GIT assays among patients with concordant (positive-positive [n = 9], negative-negative [n = 22], indeterminate-indeterminate [n = 35]) and discordant (positive-indeterminate [n = 1], negative-indeterminate [n = 7], indeterminate-negative [n = 16]) results (13.7 ± 12.1 vs. 13.5 ± 7.6 days; P = 0.958). Among 66 patients with concordant QFT-GIT results, those with persistent indeterminate results (n = 35) had higher disease severity, a lower serum albumin level and a worse in-hospital outcome compared to the others (Table 4).

### Factors associated with indeterminate QFT-GIT results

**Figure 2:**
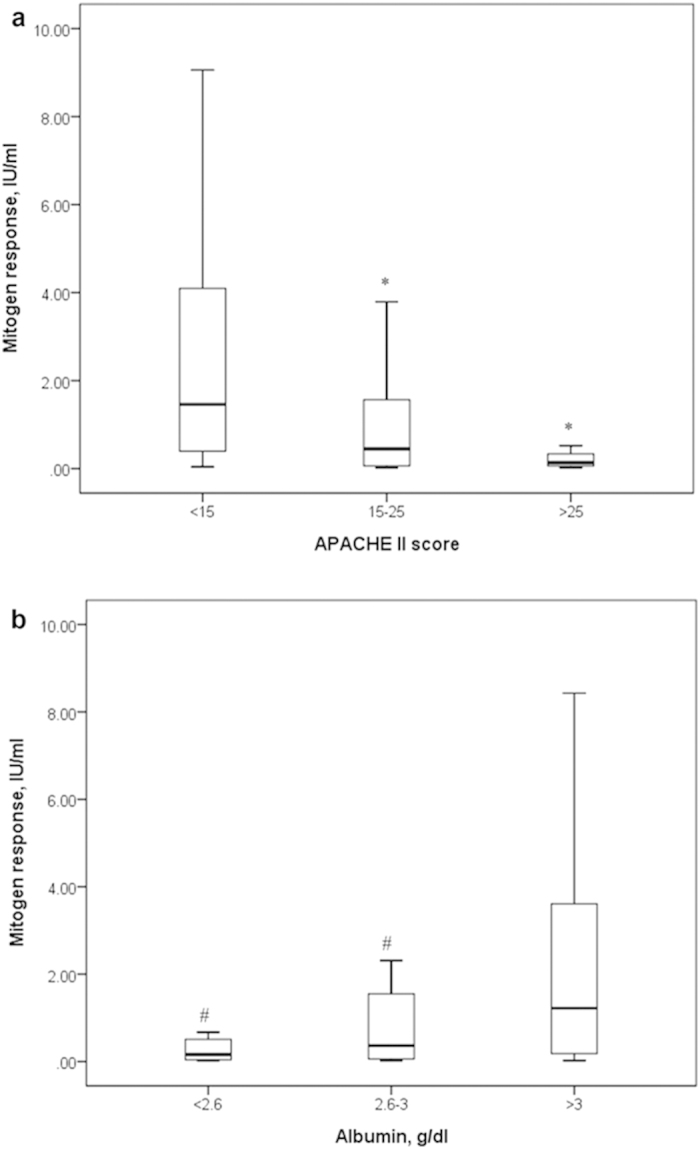
Boxplots showing the relationships between the mitogen response and the APACHE II score (a) or serum albumin value (b). *Significant difference (P < 0.05) compared with APACHE II score <15; ^#^Significant difference (P < 0.05) compared with albumin >3 g/dl. APACHE, acute physiology and chronic health evaluation; IU, international unit.

Univariate analyses indicated that an indeterminate QFT-GIT result was associated with a higher APACHE II score and a lower serum albumin value (Table 5). The multivariate logistic regression analysis confirmed that both APACHE II score (odds ratio [OR] per 1 score increment: 1.09; 95% confidence interval [CI]: 1.04–1.15) and serum albumin (OR per 1 g/dl increment: 0.32; 95% CI: 0.15–0.69) were independent variables associated with indeterminate QFT-GIT results. Given that the low mitogen response accounted for all the indeterminate results, the relationships between the mitogen response and APACHE II scores or albumin levels were explored. Figure 2a,b show that patients with an APACHE II score <15 or a serum albumin value >3 g/dl had a significantly higher mitogen response compared to other patient groups.

## Discussion

This is the first prospective study in the literature examining the IGRA response among critically ill patients. Our study shows that a high proportion (61%) of patients had indeterminate QFT-GIT results on ICU admission. Clinical characteristics associated with an indeterminate QFT-GIT result in this cohort included a higher APACHE II score and a lower serum albumin level. Moreover, APACHE II scores and albumin values were negatively and positively correlated with the mitogen response, respectively. In patients surviving to ICU discharge and having paired QFT-GIT results, no IGRA conversion or reversion was observed, but more than one third of patients had a persistent indeterminate QFT-GIT result. These findings have important implications for the use of IGRAs as diagnosing tools for LTBI or TB in the critically ill settings.

From prior studies of IGRAs in adults, widely varying proportions of indeterminate results, ranging from 0% to 41%, have been reported^22^,^23^. Numbers were discrepant on the basis of the population under study; the pooled rate of indeterminate results was 2.1% for the QFT-GIT assay, increasing to 4.4% among immunocompromised subjects^24^. An extraordinary high proportion (>20%) of indeterminate results has been observed in studies including a significant number of inpatients and HIV-infected patients^23^,^25^,^26^,^27^,^28^. Undoubtedly, immunocompromised state, particularly HIV infection, is an important predictor of an indeterminate IGRA result^29^,^30^. However, the impact of inpatient status on IGRA performance is seldom described in the literature. A recent paediatric study showed that indeterminate assays were associated with inpatient status (OR: 11.7) and the authors ascribed the causal relationship to modifiable factors, such as specimen handling, external to the patients^31^. Another study in the adult population also showed a high proportion (19.8%) of indeterminate test results among the inpatients, that was associated with host factors and preanalytical errors^28^.

In line with these two studies, a large proportion of our study subjects had indeterminate IGRA results, and it is probable that critical ill patients were too sick to mount an immune response to the mitogen challenge, as shown in prior studies^32^. The findings that a higher APACHE II score correlated with a lower mitogen response and a higher proportion of indeterminates also support our contention. Moreover, compared to previous two studies, which were retrospective in nature, our prospective study had put a lot of efforts on eliminating preanalytical and laboratory variability^24^. Thus, we believe that host factors play a major role in determining the indeterminate results in our study population. In short, the presence of acute illnesses, particularly critical ones, significantly limits the interpretability of IGRAs, and cost-effectiveness consideration and careful case selection are important parts in implementing IGRAs in this specific patient population.

Diagnosis of TB in the critically ill setting is challenging and delay in appropriate anti-TB therapy can be associated with worse ICU survival^33^. Usually, clinical symptoms, microbiological investigation and chest radiographs provide hints or evidence of TB disease. However, so-called characteristic features, such as fever, chronic productive cough and weight loss, are nonspecific among ICU patients. Acid-fast smears of respiratory samples, albeit providing rapid results, have the shortcoming of insufficient sensitivity for TB diagnosis^34^. The detection of MTB by culture, the gold standard for the definitive diagnosis of TB, takes time to yield results. In the ICU setting, studies failed to identify radiographic changes specific for TB^35^. Under such circumstances, IGRAs may be a viable modality to facilitate the diagnosis of TB. A recent meta-analysis reported a pooled sensitivity of 80% and specificity of 79% for the TB diagnosis using QFT-GIT assays, yet indeterminate results were excluded from the analysis^34^. In addition, none of the included studies specifically enrolled ICU patients. Taken together with our study showing a high percentage of indeterminate QFT-GIT results, it is advocated that the critically ill may not be appropriate candidates for IGRA testing.

In accordance with prior studies^12^,^36^, we found an association between indeterminate IGRA results and a lower serum albumin value. In critical illness conditions, hypoalbuminaemia is primarily a marker of the systemic inflammatory response that leads to protein-energy malnutrition. IGRA performance depends on intact cell-mediated immunity, especially the T-helper 1 type, that produces IFN-γ in response to the MTB-specific antigen and mitogen. Protein-energy malnutrition exerts several adverse effects on immunocompetence, including a reduction in the number and function of T cells, phagocyte dysfunction and compromised delayed cutaneous hypersensitivity^37^,^38^,^39^. Thus, it is unsurprisingly and biologically plausible that low albumin levels may increase the possibility of indeterminate results. However, it remains uncertain whether hypoalbuminaemia *per se* takes an influence on IGRA results^36^.

Besides technical factors, such as blood sampling and specimen processing, indeterminate QFT-GIT results are caused by an excess of T cell reactivity to the nil control or a limited response to the mitogen. All indeterminate results in our study were due to an inadequate mitogen response and this finding is in agreement with published experiences in the adult population^28^,^30^,^40^. An indeterminate IGRA result provides no information with regard to the likelihood of MTB infection. To date, it remains unknown what an indeterminate result means and the optimal follow-up of subjects with indeterminate results has not be established. Repeat QFT-GIT testing may be one of the choices^41^; however, in our cohort, more than two-thirds of patients with an indeterminate result and repeat testing had another indeterminate one. It is not surprising in that host factors accounting for indeterminate IGRA results in the critically ill may not be modifiable in the short run. Similarly, there are no recommendations on how to interpret repeatedly indeterminate results.

A number of limitations of the present study should be mentioned. First, in lack of a gold standard for diagnosis of LTBI, active TB disease usually serves as a surrogate. However, the limited number of TB patients in our study population makes it difficult to evaluate the diagnostic value of QFT-GIT in critically ill patients with TB. Second, our follow-up period was relatively short, *i.e.*, up to hospital discharge; thus, we are unable to realize the longer-term effects of acute critical illnesses on IGRA performance. Third, the QFT-GIT assay was the only IGRA performed in this study and it is not possible to compare QFT-GIT to the other IGRA, T-SPOT.TB (Oxford Immunotec, Oxford, UK), in the ICU settings. Finally, although we have tried hard to eliminate preanalytical and laboratory variables that may influence IGRA results, we can not exclude the possibility of some unmeasured or unknown confounders in our study. Further studies are needed to resolve these limitations.

In conclusion, we found a large proportion of indeterminate IGRA results in the ICU patients, and host factors, including the severity of critical illnesses and serum albumin levels, were associated with indeterminates. Moreover, the majority of patients with indeterminate results still did so on the follow-up testing. This study highlights the limitation in the performance of IGRAs among the critically ill. More works are required to identify best strategies to implement IGRAs and to explore undetermined factors associated with indeterminates in this particular patient population.

## Additional Information

**How to cite this article**: Huang, C.-T. *et al.* Effects of acute critical illnesses on the performance of interferon-gamma release assay. *Sci. Rep.* **6**, 19972; doi: 10.1038/srep19972 (2016).

## Figures and Tables

**Table 1:** Baseline demographics and clinical information of study subjects and their comparators.

	ICU patients (n = 118)	Diabetic patients (n = 152)
Age, years	62 ± 13	59 ± 11
Male sex	69 (59)	90 (59)
Body mass index, kg/m^2^	23 ± 5	25 ± 4
APACHE II score	21 ± 9	NA
*Mycobacterium tuberculosis* status		
* *BCG vaccination	105 (89)	132 (87)
* *History of active tuberculosis	7 (6)	4 (3)
* *Active tuberculosis	3 (3)	1 (1)
Admitting diagnoses		
* *Respiratory	57 (48)	NA
* *Gastrointestinal	16 (14)	NA
* *Cardiovascular	11 (9)	NA
* *Neurologic	11 (9)	NA
* *Genitourinary	3 (3)	NA
* *Other sepsis	12 (10)	NA
* *Others	8 (7)	NA
Comorbidities		
* *Hypertension	57 (48)	71 (47)
* *Malignancy	48 (41)	2 (1)
* *Diabetes mellitus	44 (37)	152 (100)
* *Cerebrovascular accident	23 (20)	7 (5)
* *Liver cirrhosis	16 (14)	2 (1)
* *Heart failure	15 (13)	2 (1)
* *End-stage renal disease	11 (9)	3 (2)
* *Organ transplantation	6 (5)	1 (1)
* *Autoimmune disease	6 (5)	4 (3)
Lab testing		
* *Haemoglobin, g/dl	10.5 ± 2.6	NA
* *White blood cells, k/mcl	12.6 ± 0.8	NA
* *Lymphocytes, k/mcl	1.2 ± 1.1	NA
* *Albumin, g/dl	2.8 ± 0.6	NA
* *QFT-GIT results		
* *Positive	10 (9)	35 (23)
* *Negative	36 (31)	112 (74)
* *Indeterminate	72 (61)	5 (3)
Outcomes		
* *ICU mortality	28 (24)	NA
* *Hospital mortality	43 (36)	NA

Data are presented as mean ± standard deviation or number (%).

APACHE, acute physiology and chronic health evaluation; BCG, Bacillus Calmette-Guerin; ICU, intensive care unit; NA, not applicable; QFT-GIT, QuantiFERON-TB Gold In-Tube.

**Table 2:** Comparison among patients groups with regard to the severity of critical illness.

	APACHE II < 15	APACHE II 15–25	APACHE II > 25	P value
	(n = 31)	(n = 55)	(n = 32)	
QFT-GIT results				
* *Positive	3 (10)	7 (13)	0 (0)	0.001*
* *Negative	15 (48)	16 (29)	5 (16)	
* *Indeterminate	13 (42)	32 (58)	27 (84)	
TB antigen response, IU/ml†	1.61 ± 0.99	2.78 ± 3.52	NA	0.598
Mitogen response, IU/ml	2.52 ± 2.96	1.20 ± 1.89	0.40 ± 0.65	<0.001
Nil response, IU/ml	0.08 ± 0.15	0.13 ± 0.48	0.09 ± 0.23	0.817
Outcomes				
* *ICU mortality	3 (10)	15 (27)	10 (31)	0.046*
* *Hospital mortality	7 (23)	20 (36)	16 (50)	0.024*

Data are presented as mean ± standard deviation or number (%).

APACHE, acute physiology and chronic health evaluation; ICU, intensive care unit; IU, international unit; NA, not applicable; QFT-GIT, QuantiFERON-TB Gold In-Tube; TB, tuberculosis.

^*^P for trend.

^†^Only those with a positive QFT-GIT result.

**Table 3:** Comparison of QuantiFERON-TB Gold In-Tube results on intensive care unit admission and discharge (n = 90).

QFT-GIT	ICU admission			Total
	Positive	Negative	Indeterminate	
ICU discharge				
* *Positive	9	0	0	9 (10)
* *Negative	0	22	16	38 (42)
* *Indeterminate	1	7	35	43 (48)
Total	10 (11)	29 (32)	51 (57)	90 (100)

Data are presented as number or number (%).

ICU, intensive care unit; QFT-GIT, QuantiFERON-TB Gold In-Tube.

**Table 4:** Comparison between patients with concordant and persistent indeterminate interferon-γ release assay results (n = 66).

	Paired QFT-GIT results		P value
	Positive-positive or negative-negative	Indeterminate-indeterminate	
	(n = 31)	(n = 35)	
Age, years	62 ± 13	62 ± 14	0.957
Male sex	15 (48)	20 (57)	0.477
Body mass index, kg/m^2^	24 ± 5	23 ± 4	0.333
APACHE II score			
* *<15	15 (48)	6 (17)	0.001
* *15–25	14 (45)	14 (40)	
* *>25	2 (7)	15 (43)	
Active or history of TB	2 (7)	2 (6)	1.000
Admitting diagnoses			
* *Respiratory	11 (36)	17 (49)	0.283
* *Gastrointestinal	3 (10)	6 (17)	
* *Cardiovascular	7 (23)	2 (6)	
* *Neurologic	5 (16)	4 (11)	
* *Others	5 (16)	6 (17)	
Comorbidities			
* *Hypertension	17 (55)	19 (54)	0.964
* *Malignancy	12 (39)	13 (37)	0.896
* *Diabetes mellitus	8 (26)	15 (43)	0.147
* *Cerebrovascular accident	6 (19)	9 (26)	0.538
* *Liver cirrhosis	5 (16)	5 (14)	1.000
* *Heart failure	5 (16)	5 (14)	1.000
* *End-stage renal disease	2 (7)	3 (9)	1.000
ICU events			
* *Blood transfusion	15 (48)	23 (66)	0.155
* *Use of vasopressors	10 (32)	18 (51)	0.116
* *Renal replacement therapy	5 (16)	9 (26)	0.342
Lab testing			
* *Haemoglobin, g/dl	11.2 ± 3.0	10.2 ± 2.4	0.154
* *White blood cells, k/mcl	11.2 ± 8.1	13.1 ± 6.9	0.309
* *Lymphocytes, k/mcl	1.3 ± 1.0	1.0 ± 1.0	0.247
Albumin, g/dl			
* *<2.6	6 (19)	19 (54)	0.009
* *2.6–3	10 (32)	9 (26)	
* *>3	15 (48)	7 (20)	
Hospital mortality	1 (3)	9 (26)	0.015

APACHE, acute physiology and chronic health evaluation; ICU, intensive care unit; QFT-GIT, QuantiFERON-TB Gold In-Tube; TB, tuberculosis.

**Table 5:** Factors associated with indeterminate interferon-γ release assay results.

	QFT-GIT results		P value
	Positive/Negative (n = 46)	Indeterminate (n = 72)	
Age, years	62 ± 14	62 ± 13	0.938
Male sex	25 (54)	44 (61)	0.467
Body mass index, kg/m^2^	24 ± 5	23 ± 4	0.540
APACHE II score	17 ± 8	24 ± 9	<0.001
Active TB or history of active TB	4 (9)	6 (8)	1.000
Admitting diagnoses			
* *Respiratory	22 (48)	35 (49)	0.390
* *Gastrointestinal	5 (11)	11 (15)	
* *Cardiovascular	7 (15)	4 (6)	
* *Neurologic	5 (11)	6 (8)	
* *Others	7 (15)	16 (22)	
Comorbidities			
* *Hypertension	23 (50)	34 (47)	0.768
* *Malignancy	20 (44)	28 (39)	0.621
* *Diabetes mellitus	17 (37)	27 (38)	0.953
* *Cerebrovascular accident	9 (20)	14 (19)	0.987
* *Liver cirrhosis	6 (13)	10 (14)	0.896
* *Heart failure	6 (13)	9 (13)	0.931
* *End-stage renal disease	3 (7)	8 (11)	0.525
Lab testing			
* *Haemoglobin, g/dl	10.9 ± 2.9	10.3 ± 2.4	0.212
* *White blood cells, k/mcl	12.4 ± 0.8	12.7 ± 0.8	0.869
* *Lymphocytes, k/mcl	1.3 ± 0.9	1.1 ± 1.2	0.351
* *Albumin, g/dl	3.0 ± 0.6	2.7 ± 0.5	0.002

Data are presented as mean ± standard deviation or number (%). APACHE, acute physiology and chronic health evaluation; QFT-GIT, QuantiFERON-TB Gold In-Tube; TB, tuberculosis.
